# Synthesis and performances of bio-sourced nanostructured carbon membranes elaborated by hydrothermal conversion of beer industry wastes

**DOI:** 10.1186/1556-276X-8-121

**Published:** 2013-03-07

**Authors:** Oula El Korhani, Doumit Zaouk, Sophie Cerneaux, Randa Khoury, Antonio Khoury, David Cornu

**Affiliations:** 1 Institut Européen des Membranes, UMR 5635, Ecole Nationale Supérieure de Chimie de Montpellier (ENSCM), CNRS, Université Montpellier 2, 276 rue de la Galéra, Montpellier, 34000, France; 2 Applied Physic Laboratory (LPA), Faculty of Sciences II, PR2N EDST, Lebanese University, P.O. Box 90239, Jdeidet, 1202, Lebanon; 3 Faculty of Agronomy, Lebanese University, P.O. Box 90239, Jdeidet, 1202, Lebanon

**Keywords:** Hydrothermal carbonization process, Carbon membrane, Beer waste

## Abstract

Hydrothermal carbonization (HTC) process of beer wastes (Almaza Brewery) yields a biochar and homogeneous carbon-based nanoparticles (NPs). The NPs have been used to prepare carbon membrane on commercial alumina support. Water filtration experiments evidenced the quasi-dense behavior of the membrane with no measurable water flux below an applied nitrogen pressure of 6 bar. Gas permeation tests were conducted and gave remarkable results, namely (1) the existence of a limit temperature of utilization of the membrane, which was below 100°C in our experimental conditions, (2) an evolution of the microstructure of the carbon membrane with the operating temperature that yielded to improved performances in gas separation, (3) the temperature-dependent gas permeance should follow a Knudsen diffusion mechanism, and (4) He permeance was increasing with the applied pressure, whereas N_2_ and CO_2_ permeances remained stable in the same conditions. These results yielded an enhancement of both the He/N_2_ and He/CO_2_ permselectivities with the applied pressure. These promising results made biomass-sourced HTC-processed carbon membranes encouraging candidates as ultralow-cost and sustainable membranes for gas separation applications.

## Background

In the recent years, attention has been focused on carbon-based nanomaterials to face environmental issues [1]. Mainly in the form of carbon nanotubes, these nanomaterials were advantageously used as building blocks for water filtration and gas permeation membranes, adsorbents, and environmentally friendly energy applications such as gas storage or electrodes for (bio) fuel cells [2-8]. Since 1980, carbon membranes have shown interesting performances, particularly in gas separation [9]. The chemical and physical features of carbon nanomaterials experimentally depend on the raw materials and on the preparation process. In a global and integrated sustainable route, biomass can be advantageously used as a carbon source [2,5,10-18]. These raw materials obviously represent a very low-cost alternative and possible high availability compared to petrol-derived commercial carbon sources such as classical polymeric precursors: polyacrylonitrile, polyamide and its derivatives, and cellulose [9,16]. In order to convert biomass (carbohydrate) into carbon materials, two main routes can be used, namely a pyrolysis approach or a hydrothermal carbonization (HTC) [10-12,19,20]. The HTC process can provide carbon materials with low energy consumption (<350°C) and with limited environmental impacts due to the non-generation of CO_2_ during conversion reactions. The HTC process is usually performed in a sealed autoclave and in the presence of water [10-12]. In 1913, Bergius has done pioneer works on cellulose conversion to carbon materials. The process he developed was thus extended to various carbon sources like carbohydrates such as glucose [10,12,21]. In a similar field, Antonietti et al. have performed pioneer works by elaborating a variety of carbon-based microstructures and nanostructures from hard or soft sources such as orange peels, oak leaves, pine cones, pine needles, and rice [10-12,17,22,23].

In the present study, our aim was to produce ultralow-cost membranes by sustainable routes to answer environmental issues (water and air filtrations) affecting some emerging and third countries, such as Lebanon. The strategy we developed is based on the valorization of natural products and food industry by-products. We develop a process based on the hydrothermal carbonization of Lebanese beer wastes to produce carbon-based nanoparticles (NPs). The obtained NPs were then used to produce carbon membranes of which performances in water filtration and gas separation will be presented and discussed.

## Methods

### Synthesis of carbon-based nanoparticles by hydrothermal carbonization

Carbon nanoparticles were synthesized from beer wastes by a hydrothermal carbonization process. Beer wastes were obtained from Almaza Brewery (Heineken International, Beirut, Lebanon), rated as the first brewery in Lebanon since 1933. The wastes were collected after the filtration process of beer mixture and are essentially composed of malt, water, and yeast. After drying at 100°C for 14 h, the ensuing solid was ground in a ball miller for 4 h at 200 rpm. Citric acid (Sigma-Aldrich Co., Dorset, England, UK) was used as an activating agent in the carbonization reaction [16].

The reaction was carried out in a non-stirred, 300-mL capacity Teflon-lined stainless steel autoclave (Parr Instrument Company, Moline, Illinois, USA), in which the temperature is controlled by a thermocouple (Eurotherm regulator, Invensys Eurotherm, Ashburn, VI, USA). During heating and due to experimental setup limitation, the temperature cannot exceed 350°C and the pressure of 200 bar. In a typical experiment, 15 g of beer wastes was dispersed in 120 mL of pure water for 30 min, and then, 30 mg of citric acid was added as an activating agent for the carbonization reactions occurring during the HTC process. The autoclave was sealed and heated up to 220°C for 16 h, these operating conditions being similar to HTC biomass conversion [19]. After the HTC process, the crude product contained a precipitate, the biochar (or hydrochar) and a colloidal solution, which were easily separated by centrifugation (8,000 rpm; 30 min). The charcoal was washed several times with water (18 mΩ) and then dried in an oven at 80°C for 12 h before further characterization. The colloidal solution was used as obtained. Alternatively, the colloids were destabilized by the addition of ammonia solution (1 M) up to pH of approximately 9 to yield the formation of a precipitate by colloid aggregation. The solid was filtered off on a 0.45-μm micrometric filter (Whatman, Maidstone, UK) and washed several times with water (18 mΩ), and then dried in an oven at 80°C for 2 h. For experimentation of the HTC process, it is necessary to keep in mind that for security reasons, starting solution should not exceed two thirds of the total autoclave volume.

### Carbon membrane preparation

In order to prepare porous carbon membranes, the obtained colloidal solution was concentrated at 70% (*v*/*v*) and then deposited on the inner surface of low-ultrafiltration tubular alumina membranes (ES 1426, 5 nm, Pall Membralox, NY, USA) by slip casting. The obtained membrane was treated in a tubular oven under a nitrogen atmosphere up to 1,000°C (120°C/h up to 500°C (1-h dwell) then 180°C/h up to 1,000°C (3-h dwell)).

### Characterization techniques

The soluble fraction of the used beer waste was characterized by ^1^H nuclear magnetic resonance (NMR) using a Bruker Advance 300-MHz NMR spectrometer (Madison, WI, USA), using D_2_O as reference solvent. Infrared (IR) spectroscopy was performed using the KBr pellet technique using a Spectrum Nicolet 710 Fourier-transformed IR (FTIR) apparatus in the range 4,000 to 450 cm^-1^. Raman spectrum was recorded at room temperature with an Ar-Kr laser LabRAM 1B spectrometer (HORIBA, Ltd., Kyoto, Japan). The morphology of carbon particles was observed by scanning electron microscopy (SEM) using a HITACHI S4800 microscope (Chiyoda-ku, Japan). Transmission electron microscopy (TEM) was used for deep investigation of the nanoparticles produced. This was carried out using a Philips CM20 microscope (Amsterdam, The Netherlands) operating at 200 KV at a resolution of 1.4 Å. Carbon membranes were analyzed by nitrogen adsorption-desorption isotherm using BET techniques (ASAP 2012, Micromeritics, Norcross, GA, USA) in order to identify the specific surface area of the membrane and estimate the pore diameters. Scanning electron microscopy was also used to visualize the morphology and the thickness of the elaborated carbon layer. The water filtration experiments were conducted on a home-made filtration pilot. The dynamic gas permeation test was performed on a classical separation pilot using N_2_, He, and CO_2_.

## Results and discussion

### Beer waste characterization

**Figure 1:**
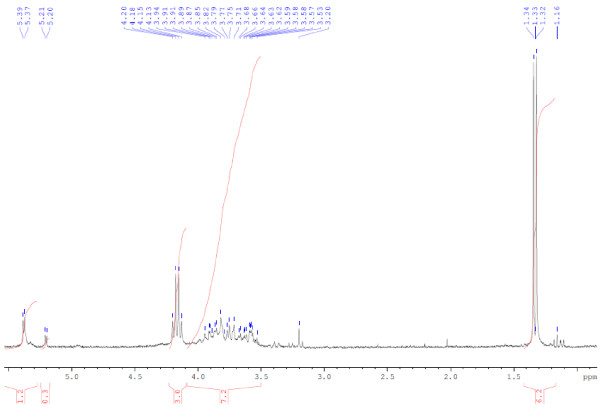
^**1**^**H NMR spectrum of the fraction of solid beer wastes soluble in D**_**2**_**O.**

The solid beer waste sample, generated by filtration just before the filling step of the beer bottles, was directly collected from the Almaza Brewery in Lebanon. The soluble fraction of waste in D_2_O was analyzed by ^1^H NMR (Figure 1). The signals around 1.3 ppm are attributed to lipidic protons and the signals between 3.0 and 4.5 ppm to carbohydrate ones [24]. This analysis is in agreement with the reported composition of beer waste [25,26].

### Carbon nanoparticles preparation and characterization

A suspension of beer wastes particles in aqueous citric acid was used as starting solution for the hydrothermal carbonization process. After reaction, the solid charcoal was separated from a colloidal solution by centrifugation. For analysis purposes, the carbon-based nanoparticles were precipitated upon aggregation by addition of ammonia solution (1 M) up to pH of approximately 9.

#### Morphological characterization of the nanoparticles

**Figure 2:**
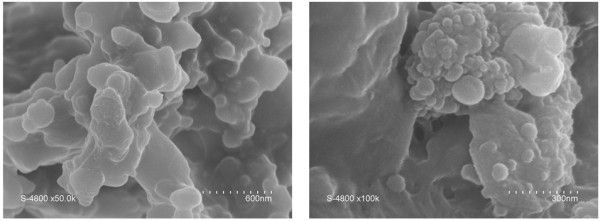
SEM images of the biochar obtained by HTC conversion of beer waste.

The carbon-based solid and nanoparticles were first observed by scanning electron microscopy and/or transmission electron microscopy in order to determine their morphology. Figure 2 shows the SEM images of the hydrochar produced by the HTC process. It can be seen that the particles are micrometric to millimetric in sizes, highly heterogeneous, and partially nanostructured in surface. This structure is presumably mimicking the one of the biomass before carbonization.

**Figure 3:**
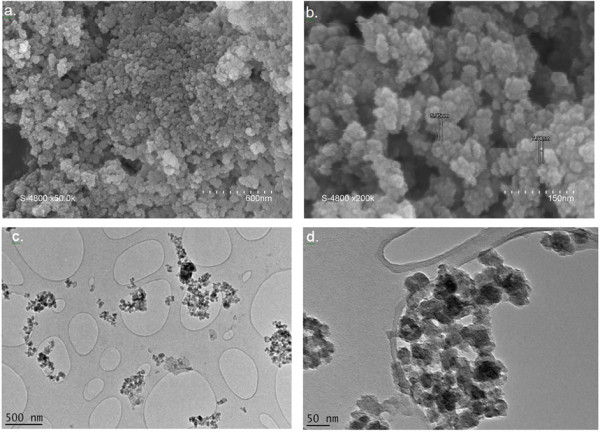
SEM (a, b) and TEM (c, d) images of carbon-based nanoparticles generated by the HTC process.

In contrast, the solid collected by destabilization of the colloid solutions is composed of agglomerated nanoparticles (Figure 3). Figure 3a,b shows field emission gun-SEM images of the as-obtained solid. The lowest quality of the image Figure 3b collected at higher magnification is due to the sample preparation procedure that did not contain any metallization step. However, this magnification allows the observation of the particle diameter with an improved accuracy. The nanoparticles exhibit a homogeneous size distribution, between 5 and 9 nm. Figure 3c,d shows typical TEM images of the nanoparticles. It is interesting to notice that the TEM grids were prepared from ethanol suspension of nanoparticles. The TEM analysis clearly underlines therefore that the agglomeration process obtained by ammonia addition is completely reversible. The morphology of these nanoparticles is very similar to the one reported for the particles obtained by HTC conversion of glucose [10,19,20].

#### Chemical characterization

**Figure 4:**
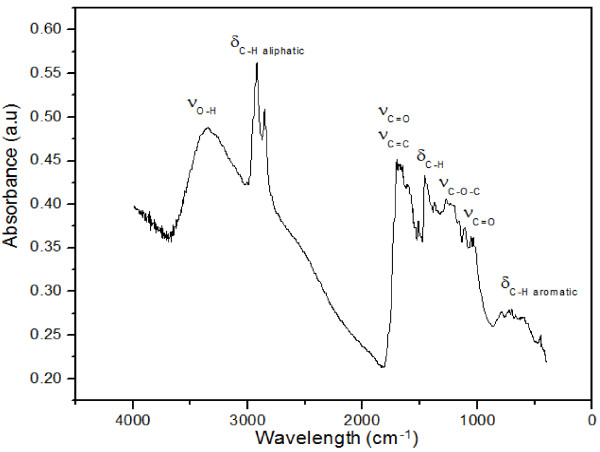
FTIR spectrum of beer-waste-derived biochar obtained by the HTC process.

The biochar and nanoparticles were analyzed by FTIR spectroscopy. Figure 4 shows typical infrared spectrum of dried biochar. By comparison with references from the literature, different stretching and vibration bands were attributed (see Figure 4) [11,18,19]. As a result, the crude biochar is obviously not fully mineralized and contains a large amount of lipid groups and some carbohydrates.

**Figure 5:**
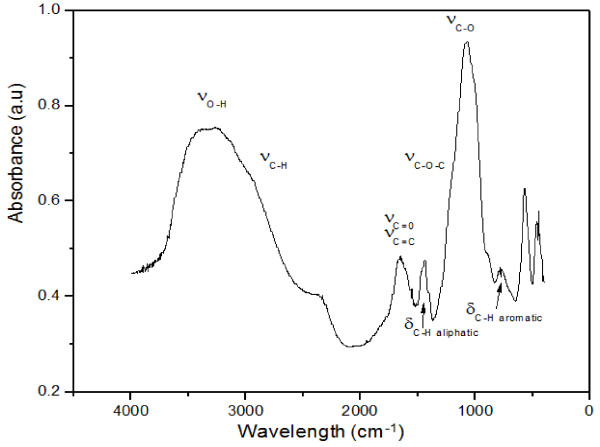
FTIR spectrum of beer-waste-derived nanoparticles obtained by the HTC process.

In contrast, Figure 5 shows a typical FTIR spectrum of nanoparticles. Important differences with the infrared spectrum of the biochar can be noticed. Similar bands have been detected, underlining the common origin of these two products. However, the signals corresponding to the carbohydrates (OH, C-O, and C-O-C vibrations) are significantly more intense in this spectrum. The nanoparticles contain therefore a more important proportion of carbohydrates to lipids than the corresponding biochar. We assume therefore that the fraction of carbohydrates, in water suspension during the HTC process, plays a key role in the formation of the nanoparticles. Further experiments will be conducted in order to collect experimental evidences for confirming or refuting this hypothesis.

**Figure 6:**
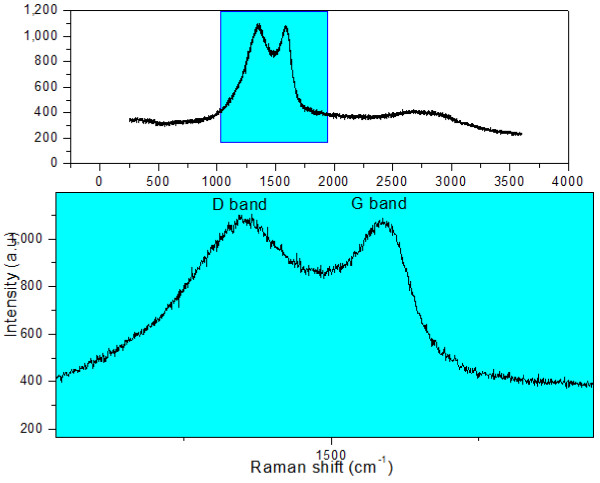
Raman spectrum of biochar produced by the HTC process.

Biochar and nanoparticles were analyzed by Raman spectroscopy. Spectra for polycrystalline graphite usually show a narrow G peak (approximately 1,580 cm^-1^) attributed to in-plane vibrations of crystalline graphite, and a smaller D peak (approximately 1,360 cm^-1^) attributed to disordered amorphous carbon [11]. As shown in Figure 6, the two peaks featuring amorphous carbon (D, 1,360 cm^-1^) and crystalline graphite (G, 1,587 cm^-1^) are present, but their relative intensity is different than in polycrystalline graphite. This result is in good agreement with works conducted on other nanoshaped carbons like nanopearls [27] and nanospheres [20].

**Figure 7:**
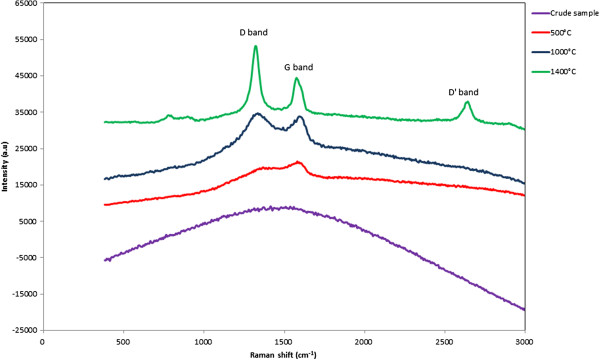
Raman spectra of the nanoparticles, crude sample, and after carbonization under nitrogen up to 1,400°C.

The Raman spectrum recorded for the nanoparticles did not show any peaks. This result was also obtained by other groups on nanoshaped carbons [19,20]. It was attributed to the fraction of graphitized carbon inside the nanoparticles which is too low to gain any significant signal. These authors used silver nanoparticles and surface-enhanced Raman scattering effect to overcome this drawback. We had a different approach by carbonizing the nanoparticles under nitrogen up to 1,400°C. The expected effect was to increase the ratio between the graphitized part of the nanoparticles and the non-mineral surface region. The different Raman spectra are presented in Figure 7. It is important to notice that the same amount of matter was analyzed during these different experiments. It is obvious that an increase of the heating temperature of the nanoparticles induces an improvement in the collected Raman signal. On the spectrum recorded for nanoparticles fired at 1,400°C, the D, G, and D’ bands were clearly identified. The relative ratio between these three peaks clearly shows the large amount of defects in the nanoparticles.

### Carbon membrane preparation and characterization

**Figure 8:**
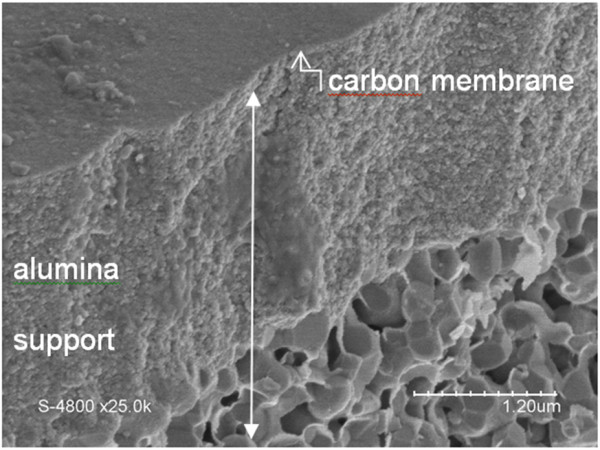
SEM images of the section (cross-sectional view) of the carbon membrane derived from beer wastes.

**Figure 9:**
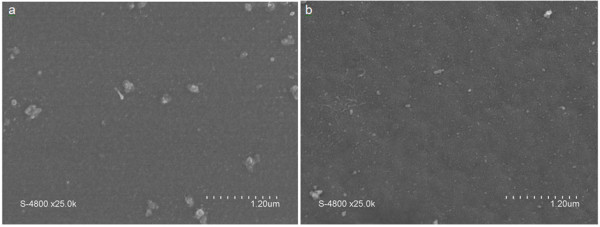
**SEM images of the membrane surface.** These were taken before (**a**) and after (**b**) heating up at 200°C during gas permeance measurements.

A carbon membrane was elaborated from the as-obtained colloidal solution, using the slip-casting technique. The agglomerated nanoparticle layer formed after deposition on the inner surface of commercial tubular alumina support was heated under argon for 2 h at 1,000°C for consolidation purposes. The formation of the carbon-based membrane was easily and visually detected by the formation of a glossy black inner surface. Figure 8 shows the SEM image of the membrane deposited on the asymmetric alumina support (cross-sectional view). The gray coloration of the alumina below the carbon layer clearly indicates the partial infiltration of colloids inside the support during the slip-casting process. The membrane exhibits a homogeneous thickness of about 50 nm. The surface appears to be rough, remembering its colloidal origin (see also Figure 9). Some particles are also observable on the surface of the layer, which were presumably generated upon breaking the membrane and support system.

**Figure 10:**
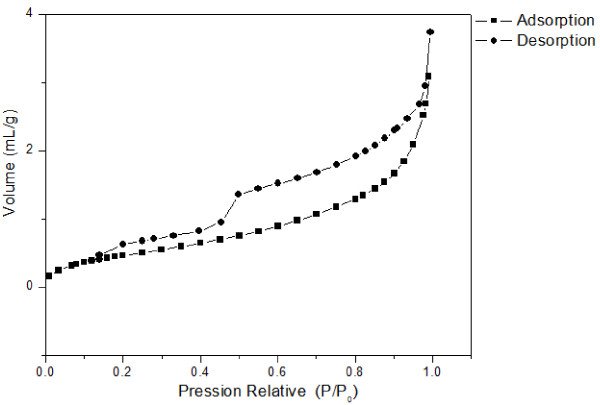
**N**_**2**_**adsorption/desorption isotherm of the HTC-processed carbon membrane.**

The N_2_ adsorption/desorption isotherm was recorded for the membrane and support system (Figure 10). For that purpose, the alumina support was sanded in order to reveal the contribution of the carbon layer. This curve clearly shows a hysteresis loop featuring the mesoporosity of the layer. This analysis, in the BET approximation, yields a pore diameter of approximately 3.6 nm (low mesoporosity). However, it is not possible to determine if this measured porosity is only due to the presence of the porous carbon membrane or partially due to the residual alumina support not totally discarded by sanding. We decided therefore to conduct dynamic water and gas separation measurements.

**Figure 11:**
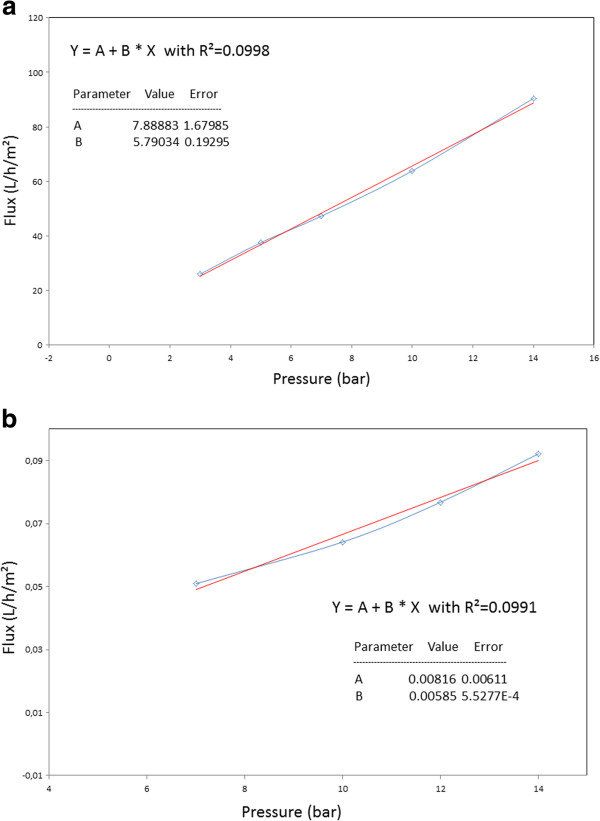
**Water flux as a function of the applied pressure for the different membranes.** (**a**) The starting alumina nanofiltration membrane and (**b**) the carbon membranes.

For a further dynamic characterization of the carbon membrane, water permeability has been measured by recording the water flux through the membrane as a function of the applied nitrogen pressure on the feed solution at room temperature. Figure 11a shows the water flux through the commercial alumina support as a function of the applied pressure, in the range of 3–15 bars. As expected, we obtained an almost linear evolution in which values are in good agreement with the ones reported by the manufacturer. In Figure 11b, the water flux through the carbon membrane deposited on alumina nanofiltration support is evidenced.

As illustrated in Figure 11b, no water flux was measured with carbon membranes below 6 bar of applied nitrogen pressure. The measured permeability is 0.005 L h^-1^·m^-2^·bar^-1^, a value which is 1,000 lower than the commercial alumina system. This result can be interpreted as a very low porous volume accessible inside the membrane. Therefore, this membrane cannot be used for water filtration applications. The quasi-dense behavior of the carbon membrane for low applied external pressure emboldens us to evaluate this material for gas separation.

**Figure 12:**
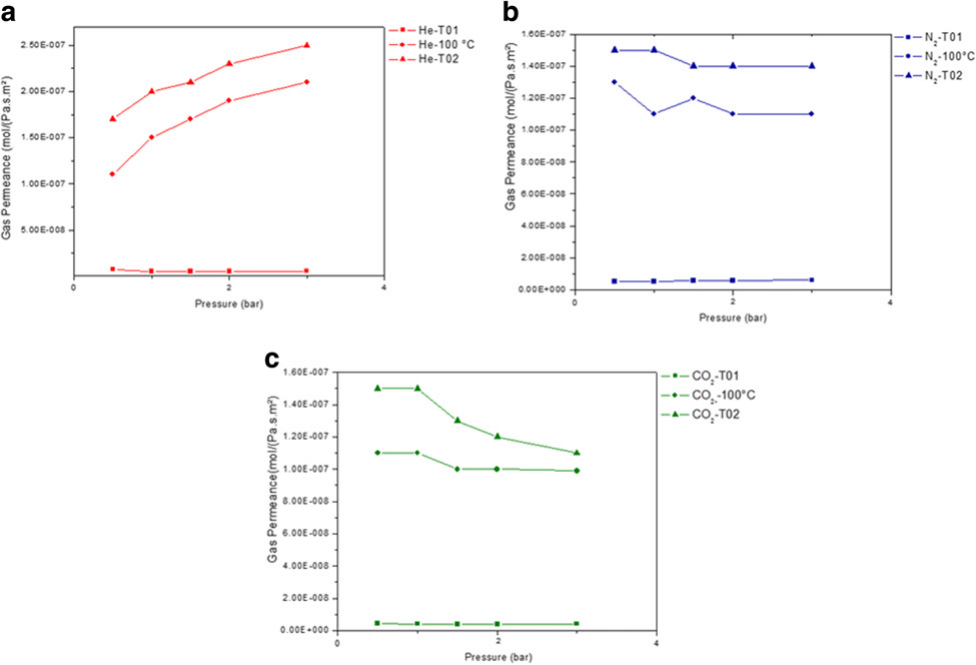
**Permeances of (a) helium, (b) nitrogen, (c) carbon dioxide as a function of the differential pressure.** These were taken at different temperatures: 25°C (T01), 100°C, and 25°C after an exposure of up to 100°C (T02).

**Figure 13:**
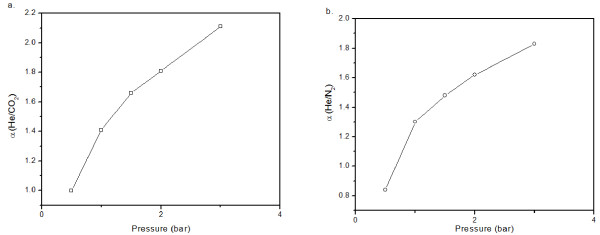
**He/CO**_**2**_**selectivity (a) and He/N**_**2**_**selectivity (b) as a function of the applied pressure at 100°C.**

From the 1970s, carbon membranes have been extensively used for gas separation [6,9,28]. Different studies were conducted on membranes originating from different sources such as polymers and carbohydrates (glucose) and have demonstrated promising permeance values in the range of 10^-8^ to 10^-9^ mol·m^-2^·Pa^-1^·s^-1^, associated with high selectivity. For instance, a carbon membrane elaborated by pyrolysis of commercial polymers and having a pore diameter between 3 and 5 Å has demonstrated a He/CO_2_ selectivity of 4, and a He/N_2_ selectivity between 20 and 40 [9]. In our case, the gas separation test was driven using three types of gases, namely helium (He, kinetic diameter = 2.6 Å), carbon dioxide (CO_2_, kinetic diameter = 3.3 Å), and nitrogen (N_2_, kinetic diameter = 3.64 Å). The permeances of these gases were recorded as a function of the pressure at different temperatures of 25°C (T01), 100°C, and after cooling down again to 25°C (T02) (Figure 12). At 25°C, the membrane gave a 10^-9^ mol·m^-2^·Pa^-1^·s^-1^ permeance value for He, CO_2_, and N_2_, which is in good agreement with the values reported in the literature [6]. At 100°C, a stable flux was obtained exhibiting a permeance in the range of 10^-7^ mol·m^-2^·Pa^-1^·s^-1^. We also observed an increase in the permeance while increasing the temperature up to 100°C whatever the used gas and the applied pressure were [26]. We assume that this result may reflect a Knudsen diffusion mechanism for the gas separation. For CO_2_ and N_2_, these systems enter into an apparent stationary regime by varying the pressure, and their permeances appear to become almost constant whatever the applied pressure was. In contrast, the permeance of helium increases with the applied pressure (lower kinetic diameter). As a consequence, the selectivity of helium versus the other gases increases with the pressure up to approximately 2 (Figure 13). This value is lower than the one reported in the literature [6].

**Figure 14:**
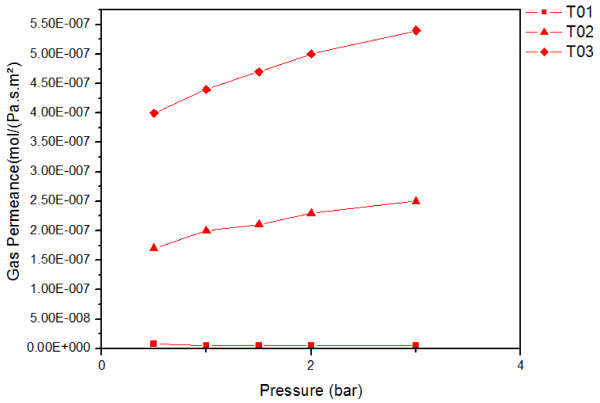
**Permeances of helium at different temperatures using the same membrane.** Permeances at 25°C (T01), at 25°C but after an exposure at 100°C (T02), and the same membrane after an exposure at 200°C (T03).

After measurement at 100°C, the membrane was cooled down to 25°C, and its permeance was measured again for each gas (Figure 12). By comparing T01 and T02, we have observed a significant increase of the permeances (by a 10^2^ factor) whatever the studied gas was. By considering this result, we underwent measurements at 200°C. At this temperature, it was not possible to obtain a stabilized flux for any studied gas and therefore to measure any permeance value. After cooling down to 25°C, we have measured again the permeance using helium (Figure 14). As illustrated by this figure, the permeance of the carbon membrane towards helium is increased after the membrane was exposed to higher operating-temperature conditions. Our assumption is that the membrane underwent a microstructural evolution during the high-temperature measurement. In order to confirm the latter, the membrane surface was analyzed by SEM after the experiment, done at 200°C (Figure 14). We can clearly conclude from the images of Figure 9 that the surface of the membrane underwent a microstructural evolution upon heating which yielded to an increase of its surface roughness. Fracture surface view analysis did not reveal any significant evolution of the membrane thickness.

## Conclusions

Hydrothermal carbonization process of beer wastes (Almaza Brewery) yields a biochar and homogeneous carbon-based nanoparticles (NPs). Carbohydrates, released by the wastes in water, are supposed to play a role in the formation mechanism of the NPs, and further experiments will be driven in the future to elucidate the latter. The NPs have been used to prepare carbon membrane on commercial alumina support. As evidenced in water filtration experiments, there is a quasi-dense behavior of the membrane with no measurable water flux below an applied pressure of 6 bar. Gas permeation tests were conducted and gave remarkable results: (1) the existence of a limit temperature of utilization of the membrane is below 100°C in our experimental conditions; (2) an evolution of the microstructure of the carbon membrane with the operating temperature yielded to improvement in its gas separation performances; (3) the permeance of the gas is temperature dependent and should be driven by a Knudsen diffusion mechanism; and (4) the He permeance is increasing with the applied pressure in entrance on the system, whereas N_2_ and C0_2_ permeances are stabilizing in the same conditions. This result yields an increase of the selectivity He/N_2_ and He/CO_2_ with the applied pressure. The obtained selectivity values are below the ones reported in the literature but further experiments are in progress in order to improve this value by optimizing the membrane microstructure and porosity. These promising results made biomass-sourced HTC-processed carbon membranes promising candidates as ultralow-cost and sustainable membranes for gas separation applications. Since He exhibits a kinetic diameter closed to that of H_2_, applications as membrane for H_2_ separation can be envisaged, for instance, for fuel cell applications.

## Competing interests

The authors declare that they have no competing interests.

## Authors’ contributions

OEK carried out the membrane preparation and characterization. DZ participated in the membrane chemical characterization. SC participated in the membrane dynamic characterization. RK participated in the beer-waste hydrothermal conversion. AK participated in the membrane chemical characterization. DC participated in the membrane preparation and characterization and drafted the manuscript. All authors read and approved the final manuscript.
